# No Association between Elevated Thyroid-Stimulating Hormone at Birth and Parent-Reported Problem Behavior at Preschool Age

**DOI:** 10.3389/fendo.2016.00161

**Published:** 2016-12-19

**Authors:** Caroline Trumpff, Jean De Schepper, Johan Vanderfaeillie, Nathalie Vercruysse, Jean Tafforeau, Herman Van Oyen, Stefanie Vandevijvere

**Affiliations:** ^1^Unit of Public Health and Surveillance, Scientific Institute of Public Health, Brussels, Belgium; ^2^Faculty of Psychology and Educational Sciences, Vrije Universiteit Brussel, Brussels, Belgium; ^3^Faculty of Psychology and Educational Sciences, Université Libre de Bruxelles, Brussels, Belgium; ^4^Department of Paediatric Endocrinology, UZ Brussel, Vrije Universiteit Brussel, Brussels, Belgium

**Keywords:** behavioral problems, thyroid-stimulating hormone, preschool children, iodine deficiency, pregnancy

## Abstract

**Objectives:**

Mild level of iodine deficiency during pregnancy may reduce maternal thyroid hormone production and supply to the fetus hence affecting brain neurodevelopment. The aim of the present study was to investigate the association between elevated neonatal thyroid-stimulating hormone (TSH) level (>5 mU/L), used as a marker of maternal mild iodine deficiency during late pregnancy, and behavioral development of preschool children.

**Methods:**

This retrospective cohort study included 310 Belgian mothers and their children aged 4–5 years old with TSH levels in the range of 0.45–15 mU/L at birth. The TSH level was measured in dried blood spots on filter paper collected by heel stick 3–5 days after birth. Low birth weight, prematurely born children, or children with congenital hypothyroidism were excluded. The degree of behavioral problems was evaluated using the *Child Behavior Check List (CBCL) for age 1½–5 years* questionnaire. Relevant socioeconomic, maternal, and child factors were also collected.

**Results:**

TSH concentrations and CBCL scores were not associated both in univariate analysis and when adjusting for confounding factors in multivariate analysis.

**Discussion:**

Elevated TSH concentrations measured at birth was not associated with behavioral development scores.

## Introduction

Iodine is essential for the synthesis of thyroid hormones that are involved in many functions including brain development (1, 2). While severe iodine deficiency is disappearing worldwide, mild iodine deficiency (MID) is still present in many developed countries (3).

Pregnant women are at a higher risk of iodine deficiency because their nutritional iodine requirements are almost doubled due to physiological changes. Thyroid hormones are involved in several important steps of brain development including cell migration and differentiation (4). A sufficient transfer of maternal thyroid hormones to the fetal brain is necessary from the early stage of pregnancy, and a lack of thyroid hormones during pregnancy may disturb the brain development of the fetus. Even MID can lead to mild thyroid dysfunction that may affect the fetal brain development (5). Insufficient iodine stock can lead to maternal hypothyroxinemia that is a common condition in pregnancy characterized by a low level of free thyroxin with thyroid-stimulating hormone (TSH) within the normal range (6). Several studies have shown that maternal hypothyroxinemia can lead to impaired cognitive and psychomotor development in offspring (5, 7).

Little is known about the impact of mild thyroid dysfunction during pregnancy on behavioral development of children. A prospective study conducted in an geographic area of moderate iodine deficiency in Italy found that children of hypothyroxemic mothers had an increased risk of developing attention deficit hyperactivity disorder (ADHD) symptomatology (8). However, that study was conducted on a very limited size sample. Two larger researches have been conducted in order to investigate the effect of maternal hypothyroxinemia on behavioral development of the offspring. One of these found a relationship between higher levels of maternal TSH and higher externalizing scores in children aged 1½ and 3 years old, while no association was found between maternal fT4 and total T4 and behavioral problem scores in children (9). In contrast, the second study found that maternal hypothyroxineamia was linked to an increased risk of showing ADHD symptoms at 8 years old (10).

Another study found that MID during pregnancy, measured by urinary iodine excretion, was related in children of 4 years of age to impaired executive functioning (11), impairments that are known to be related to behavioral problems such as ADHD, oppositional defiant disorder, or conduct disorder (11).

Put together, these data indicate a potential implication of maternal and/or fetal thyroid dysfunction in the vulnerability of children to behavioral problems. However, the number of studies is limited and their results are controversial, thereby requiring further studies.

Thyroid-stimulating hormone concentration in whole blood measured at birth has been proposed as an indicator of maternal iodine status during late pregnancy (12–14). When thyroid disease is excluded, the thyroid hormone level is mainly dependent on body iodine stocks provided by nutrition. TSH is liberated in the bloodstream to stimulate the production of thyroid hormones when they are lacking. When their amount is adapted, TSH secretion is stopped by a negative feedback loop. In cases of iodine deficiency, iodine stocks are insufficient to produce thyroid hormones, and the TSH concentration increases in the bloodstream. Following this logic, an increased concentration can be used as an indicator of iodine deficiency. The world health organization (WHO) has proposed to use the TSH results provided by screening programs for congenital hypothyroidism as an index for the evaluation of iodine status of the population (12). A proportion of TSH results with a concentration above 5 mU/L below 3% was proposed as an indicator of iodine sufficiency, between 3 and 19.9% of MID, between 20 and 39.9% of moderate iodine deficiency and above 40% of severe iodine deficiency (12).

Several studies showed that elevated TSH levels after birth were associated with suboptimal cognitive and psychomotor outcomes (15–19). However, some factors other than iodine deficiency can increase TSH concentration at birth such as timing of testing, mothers or newborn health condition, and drug intake (20, 21). We previously reported no association between elevated TSH measured at birth and cognitive and psychomotor development of preschool children when confounding factors were taken into account (22, 23).

To our knowledge, the association between elevated neonatal TSH and behavioral problems has never been investigated.

The aim of this study was to investigate the association between elevated TSH concentration at birth, used as a marker of MID during late pregnancy, and behavioral development of preschool-age children, taking into account potential confounding factors. We hypothesized that a higher TSH level in newborn children would be related to the childrens’ increased tendency to internalize and externalize problems, assessed using the age-adapted Child Behavior Check List questionnaire.

## Materials and Methods

### Subjects

We used data from the PsychoTSH study, a Belgian retrospective cohort study that has been previously described in detail (22–24). In total, 310 children aged 4–5 years old with a neonatal TSH concentration in the range 0.45–15 mU/L were included in the present study. Neonatal TSH data were provided by the Brussels newborn screening center for metabolic disorders [Laboratoire de Pédiatrie, Université Libre de Bruxelles (ULB), Brussels]. The selection of children was performed from the total samples of, respectively, 29,013, 29,602, and 30,126 neonates, screened in 2008, 2009, and 2010. Children were stratified by sex and TSH level: for each sex and TSH interval, 19 children were selected randomly (0–1, 1–2, 2–3, 3–4, 4–5, 5–6, 6–7, 7–8, 8–9, and 9–15 mU/L). The day of collection was taken into account for the selection, only children with sample collected 3–5 days after birth were included. Exclusion criteria were (1) congenital hypothyroidism (15 mU/L or higher), (2) prematurity (<37 weeks), and (3) low birth weight (<2,500 g). Children with neurological disease and/or born from multiple birth were excluded during the recruitment procedure. The sample size of the TSH study was calculated for the relationship between TSH and cognitive score (22, 24).

### Ethics and Privacy Commission

Before the start of the procedure, a written informed consent was obtained from the parents of the children. The *Ethical Committee of the Erasmus Hospital* (Université Libre de Bruxelles, Brussels, Reference CCB: B40620109191) in accordance with the Code of Ethics of the World Medical Association for experiments involving humans (Declaration of Helsinki) gave the ethical approval of the study. The study was also approved by the *Belgian Privacy Commission* (Reference: RN 29/2012).

### TSH Measurements

Neonatal TSH level was measured in dried blood spots on filter paper collected by a heel stick 3–5 days after birth using a time-resolved fluoroimmunoassay (Autodelfia method) (25). The reproducibility of the TSH values was tested in the range 0–15 mU/L. In order to determine the coefficient of variation, TSH was analyzed twice at 50 different TSH values. For the TSH values ranging from 0.9 to 15 mU/L, the coefficient of variation was below 20%. For the TSH values below 0.9 mU/L, a TSH value of 0.45 mU/L was used in the statistical analysis.

### Urinary Iodine Concentration (UIC) Assessment

A sample of urine was collected from the child during the home visit (at 4–5 years) to determine the current iodine status among the children included in the study. The urine samples were frozen at −80°C until analysis. Urinary iodine concentration (UIC) was measured using a modification of the Sandell–Kolthoff reaction with spectrophotometric detection (26) at the Erasmus Hospital.

### Anthropometric Measurements Methodology

During the procedure, body weight, height, and child head circumference were measured using SECA 815 and SECA 804 scales, a SECA 214 stadiometer and a flexible tape measure SECA 212.

### Psychosocial Assessment

Psychosocial development, including behavioral and emotional problems, was assessed using the French version of the “Child Behavior Check List (CBCL) for ages 1½–5 years.” This standardized questionnaire was developed by Achenbach assessing social competence, behavioral, and emotional problems in children aged 1½–5 years old (27). The CBCL/1½–5 has a good reliability and validity (27). The syndrome scales has been shown to have an acceptable to good fit in 23 studies across diverse societies (28). The internal consistency of internalizing and externalizing syndrome scales has been tested previously and Cronbach’s alpha vary between 0.88 and 0.92 (29). The Child Behavior Checklist has been used previously in preschool children to assess the effect of maternal thyroid dysfunction on behavioral problem (7, 9) or the effect of subclinical hypothyroidism on psychosocial development (30).

The CBCL questionnaire was filled out by mothers during a home visit. It enables the rating of problematic behaviors based on a description of the child’s functioning for the last two months. The CBCL enables the calculation of 3 scores: a total problem score, a score on internalizing problems, and a score on externalizing problems.

The internalizing problems score is calculated from the following four syndrome scales: emotional reactive, anxious depressed, somatic complaints, and withdrawal. The externalizing problems score is calculated from the following two syndrome scales: attention problems and aggressive behavior. The total problems score includes internalizing and externalizing scores together with the “sleep problems” syndrome scale.

In addition to those three scores, the CBCL checklist enables the calculation of the following DSM-oriented scores: affective problems, anxiety problems, pervasive developmental problems, attention deficit/hyperactivity problems, and oppositional defiant problems.

The Achenbach’s manual provides functioning cut-off points in order to differentiate between a “normal” and a “problematic” score. These cut-off points provide a normal (below 65), a borderline (between 65 and 70), and a clinical range (higher than 70) for the scores.

The ASEBA Windows software Assessment Data Manager (ADM) was used to compute the scores.

### Descriptive Variables, Covariates, and Effects Modifiers

Information about covariates and effect modifiers was retrieved from the data provided by the ULB newborn screening center for metabolic disorders, the child’s health booklet, and a self-report questionnaire filled out by the mother during the home visit.

The following information was collected about the pregnancy history and the maternal antecedents: maternal thyroid disease, drug intake, alcohol consumption and cigarette smoking before or during pregnancy, gestational diabetes and treatment of diabetes, maternal age at birth, reproductive history, parity, gravidity, pre-pregnancy body mass index (BMI), and maternal weight gain during pregnancy. The following information was collected about the delivery: type of delivery and season of birth. The following child parameters were retrieved: perinatal anoxia, health problems of the newborn, breastfeeding, chronic disease of the child, nursery school attendance, child bilingualism, and previous cognitive assessment of the child. The socioeconomic background data collected were as follows: maternal/paternal education and employment, household income, marital status, area of residence, maternal age, housing.

In addition, several questions contained in the self-reported questionnaire aimed to assess different psychological factors potentially influencing the mental development of the child: child negative life events, maternal mental health, maternal social support, marital discord and parent–child interactions. Maternal mental health was also assessed using the “General Health Questionnaire” items and vitality scale questionnaire of the “Short Form Health Survey” (31).

### Data Analysis

Statistical analysis was performed using SAS statistical software 9.3 (SAS Institute Inc., Cary, NC, USA) for univariate analysis and Stata version 13 (StataCorp, College Station, TX, USA) for multivariate analysis. Statistical tests were two-sided and tests with *p*-value < 0.05 were considered statistically significant.

Neonatal TSH values and UIC were presented as median, and IQR and CBCL scores were presented as mean and SD. Neonatal TSH values were classified in two groups: below 5 mU/L and higher or equal to 5 mU/L.

Univariate associations between CBCL scores and TSH were assessed using Pearson correlation. Univariate association between CBCL scores and maternal and children parameters was assessed using linear regression for continuous variables and with student’s *t*-test and ANOVA with Bonferroni correction for categorical variables. In addition, Chi square test was performed to analyze multivariate association between TSH levels (<5 mUI/L and ≥5 mUI/L) and borderline (65< and <70)/clinical score (≥70) at CBCL checklist.

Multivariate linear models were used to study the predictors of variation of CBCL scores in children. All variables associated with CBCL scores with a test *p* < 0.20 were included in the selection procedure. TSH was dichotomized using a cut-off of 5 mU/L. A stepwise backward selection procedure with a probability of entry of 0.10 and exit probability of 0.15 was used to build the final multiple linear regression models. The normality of the distribution of residuals was tested using normal plot of residuals. Linearity and homoscedasticity of residuals were checked by examination of the plot of standardized residual. Collinearity between predictors was tested using the test of variance inflation factor (VIF), and individuals’ VIF for each parameter in the model was around 1. Univariate association between the variables to be inserted in the model was tested with Pearson correlation. Variables correlated with each other were not included together in the model.

## Results

### Demographic Characteristics

Descriptive characteristics by gender of the population studied are shown in Table 1. In total, 310 children (*n* = 137 girls) aged 4 and 5 years were included in the study.

**Table 1:** **Descriptive and demographic characteristics of the study population according to gender**.

	Total		Male		Female	
	
	*N*	%	*N*	%	*N*	%
**Thyroid-stimulating hormone level at birth (mU/L)**						
0.45–1	47	15.2	26	8.4	21	6.8
2	35	11.3	21	6.8	14	4.5
3	41	13.2	23	7.4	18	5.8
4	40	12.9	20	6.5	20	6.5
5	34	11.0	19	6.1	15	4.8
6	39	12.6	20	6.5	19	6.1
7	35	11.3	21	6.8	14	4.5
8	14	4.5	10	3.2	4	1.3
9	14	4.5	6	1.9	8	2.6
10–15	11	3.6	7	2.3	4	1.3
**Age at examination**						
4 years	234	75.5	130	42.0	104	33.5
5 years	76	24.5	43	13.9	33	10.6
**Children ethnicity**						
Europe (Caucasian)	248	84.6	139	47.4	109	37.2
Asia	3	1.0	2	0.7	1	0.3
Sub-Saharan Africa	15	5.1	7	2.4	8	2.7
North Africa	27	9.2	16	5.5	11	3.8

*PsychoTSH study (*N* = 310), Belgium, 2008–2014*.

*N, number of subjects*.

### Neonatal TSH Levels at Birth and Current Iodine Status of the Studied Population

The median (range) TSH level at birth of the study sample was 3.6 mU/L [1.8–5.8 (IQR), 0.45–13.9 (min–max)]. In this sample, 197 (63.5%) children had a TSH level measured at birth lower than 5 mUI/L, and 113 (36.5%) children had a TSH higher or equal to 5 mUI/L. The median iodine concentration of the sample was 141.4 µg/L [87.9–239.9 (IQR)] indicating iodine sufficiency of the children sample.

### Univariate Association between CBCL Scores and Studied Parameters

The mean CBCL total problem score was 48.0 [10.1 (SD), 28–88 (min–max)]; the mean CBCL external problem score was 46.7 [9.5 (SD), 28–77 (min–max)]; and the mean CBCL internal problem was 49.5 [11.1 (SD), 29–86 (min–max)].

Several child, maternal, socioeconomic, and iodine status parameters were shown to be associated with CBCL scores in univariate analysis (see Tables 2–4). A negative association was found between neonatal TSH concentration, CBCL total problem score (*p* = 0.038) and internalizing problem score (0.023). No significant associations were found between DSM-oriented scores and neonatal TSH levels (data not shown).

**Table 2:** **Association of CBCL scores at preschool age with infant, maternal, and household characteristics: categorical variables**.

	Total		Total problem score			Externalizing score			Internalizing score		
	*N*	%	Mean	SD	*p*-Value^a^	Mean	SD	*p*-Value^a^	Mean	SD	*p*-Value^a^
**Children characteristics**											
Gender					0.082			**0.004**			0.381
Male	173	55.81	48.87	10.31		48.05	10.07		50.05	10.86	
Female	137	44.19	46.86	9.74		44.96	8.50		48.94	11.31	
Neonatal hospital attendance					**0.028**			0.117			**0.047**
Yes	24	8.00	52.04	12.93		49.33	10.52		53.58	12.93	
No	276	92.00	47.36	9.68		46.19	9.28		48.92	10.80	
Breastfeeding at 6 months					0.121			**0.034**			0.165
<6 months	150	48.54	47.07	9.83		45.73	9.08	*	48.64	11.11	
>6 months	123	39.81	48.06	9.46		46.55	9.09		49.71	10.59	
no breastfeeding	36	11.65	50.92	12.82		50.31	11.68	*	52.53	12.39	
Negative life event					**0.013**			0.056			**0.019**
Less than 3	267	86.41	47.38	9.76		46.22	9.27		48.97	10.92	
More than 3	42	13.59	51.55	11.49		49.24	10.56		53.29	11.46	
**Socioeconomics characteristics**											
Monthly income					**0.001**			0.054			**0.006**
<2,000 euro	38	12.67	52.70	11.92		49.22	10.21		54.14	11.97	
≥2,000 euro	262	87.33	47.06	9.71		46.02	9.31		48.71	10.92	
Mother education level					<0.0001*			0.001*			<0.0001*
No/primary	10	3.28	52.20	11.32		51.60	10.75		53.90	9.85	
Lower high school	22	7.21	56.90	10.50		51.76	9.20		58.62	10.53	
Upper high school	50	16.39	51.14	8.75		49.20	7.17		52.59	9.99	
University or higher	223	73.11	46.10	9.74		45.29	9.67		47.79	10.96	
**Maternal characteristics**											
Delivery					0.097			0.225			0.172
Normal	234	75.73	47.19	10.06		46.11	9.47		48.73	11.13	
Cesarean	43	13.92	49.16	8.91		46.95	8.76		51.86	9.93	
With vacuum	31	10.03	51.65	11.57		49.65	10.23		52.16	12.01	
With forcepts	1	0.32	43.00			40.00			45.00		
Parity—First child					**0.006**			0.158			**0.002**
Yes	132	42.72	49.77	9.48		47.52	9.00		51.78	10.29	
No	177	57.28	46.55	10.38		45.98	9.78		47.78	11.39	
Previous miscarriage					**0.012**			**0.026**			0.150
Yes	76	24.68	50.35	11.76		48.65	10.18		51.01	12.78	
No	232	75.32	47.00	9.37		45.84	9.15		48.89	10.43	
Smoking during pregnancy					**0.000**			**0.001**			**0.001**
≥ 10 cigarettes/day	6	1.94	62.83	14.16		59.00	12.41		64.33	11.04	
<10 cigarettes/day or non-smoking	304	98.06	47.69	9.81		46.44	9.32		49.27	10.87	
Alcohol during pregnancy					0.356			0.786			0.168
Non-consumer	209	68.08	48.26	9.98		46.69	9.14		50.08	10.91	
Still consuming	98	31.92	47.11	10.42		46.38	10.23		48.20	11.45	
Mother social support					**0.019**			0.098			0.133
3 or less	98	31.72	49.91	10.78		47.94	9.20		50.92	11.71	
4 or more	211	68.28	46.98	9.70		46.00	9.63		48.87	10.78	
Mother mental distress score					**<0.0001**			**0.031**			**<0.0001**
Low (<4)	259	88.10	47.16	9.83		46.17	9.36		48.86	10.87	
Elevated (>4)	35	11.90	53.69	11.62		49.89	10.67		55.89	11.31	
Mother vitality Index					0.085			0.023			0.570
Optimal vitality	269	89.07	48.25	10.18		47.15	9.45		49.50	11.32	
Suboptimal vitality	33	10.93	44.97	9.92		43.09	9.46		48.31	10.15	

*PsychoTSH study (*N* = 310), Belgium, 2008–2014*.

*A higher CBCL score is related to higher developmental problems*.

*^a^p-Value from simple t-test or ANOVA*.

*Significant with Bonferroni correction for multiple testing.

*N, number of subject*.

*Statistically significant p-Values (p < 0.05) are highlighted in bold*.

**Table 3:** **Association of CBCL scores at preschool age with infant, maternal, and household characteristics: continuous variables**.

				Total problem score			Externalizing score			Internalizing score		
				
				Pearson correlation								
				
	*N*	Median	IQR	R		*p*-Value^a^	*R*		*p*-Value^a^	*R*		*p*-Valuea
TSH (mUI/L)	310	3.6	1.8–5.9	−0.12		0.038	−0.10		0.081	−0.13		0.023

				**Univariate linear regression**								
				
	***N***	**Mean**	**SD**	***b***	**SE**	***P*^b^**	***b***	**SE**	***P*^b^**	***b***	**SE**	***P*^b^**
Term pregnancy (week)	308	39.3	1.6	−0.26	0.36	0.481	−0.25	0.34	0.473	−0.37	0.40	0.360
Birth weight (g)	310	3,394.2	429.8	0.00	0.00	0.968	0.00	0.00	0.660	0.00	0.00	0.314
Birth length (cm)	310	50.0	3.2	−0.14	0.18	0.444	−0.04	0.17	0.806	−0.27	0.20	0.187
Birth HC (cm)	269	34.6	3.1	0.21	0.19	0.267	0.18	0.18	0.334	0.18	0.21	0.389
Mother age (years)	310	36.5	5.1	−0.18	0.11	0.117	−0.07	0.11	0.498	−0.26	0.12	**0.040**

*PsychoTSH study (*N* = 310), Belgium, 2008–2014*.

*A higher CBCL score is related to higher developmental problems*.

*^a^p-Value from Pearson Correlation*.

*^b^p-Value from Univariate Linear Regression*.

*IQR, inter quartile range; HC, head circumference; N, number of subject*.

*Statistically significant p-Values (p < 0.05) are highlighted in bold*.

**Table 4:** **CBCL scores at preschool age and markers of iodine status**.

			Total problem score			Externalizing score			Internalizing score		
	*N*	%	Mean	SD	*p*-Value^a^	Mean	SD	*p*-Value^a^	Mean	SD	*p*-Value^a^
**Pregnancy and neonatal period**											
Neonatal TSH level (mU/L)					0.152			0.051			0.266
<5	197	63.55	48.60	10.30		47.49	9.66		50.09	11.19	
≥5	113	36.45	46.89	9.68		45.29	9.16		48.64	10.81	
Vitamins during pregnancy					0.191			0.286			0.139
Containing iodine	51	43.59	47.43	9.29		46.02	8.67		48.73	10.76	
No vitamins	66	56.41	49.89	10.41		47.98	10.51		51.73	10.63	
**Children characteristics**											
Urinary iodine concentration (μg/L)					0.086			0.116			0.098
<100	82	30.37	48.37	9.90		47.18	9.20		49.90	10.93	
100–149	67	24.81	49.12	10.69		47.98	9.79		50.65	11.96	
150–294	60	22.22	46.36	9.28		44.59	9.27		49.27	10.27	
≥250	61	22.59	45.11	9.42		44.92	9.57		46.11	10.08	
Household salt					0.189			0.092			0.356
Iodinised salt	96	34.53	47.39	9.77		46.42	9.63		48.82	10.91	
Non-iodinised salt	170	61.15	47.63	10.11		45.99	9.31		49.40	11.05	
No salt	12	4.32	53.00	13.64		52.25	11.89		53.75	14.72	
Child dietary supplement intake					**0.004**			**0.004**			**0.025**
Yes	196	65.55	46.71	9.54		45.54	9.12		48.47	10.71	
No	103	34.45	50.26	10.60		48.84	9.87		51.50	11.47	

*PsychoTSH study (*N* = 310), Belgium, 2008–2014*.

*A higher CBCL score is related to higher developmental problems*.

*^a^p-Value from simple t-test or ANOVA*.

*Significant with Bonferroni correction for multiple testing.

*N, number of subject*.

*Statistically significant p-Values (p < 0.05) are highlighted in bold*.

### Neontal TSH Concentration and Severity Rating at CBCL Scores

**Figure 1:**
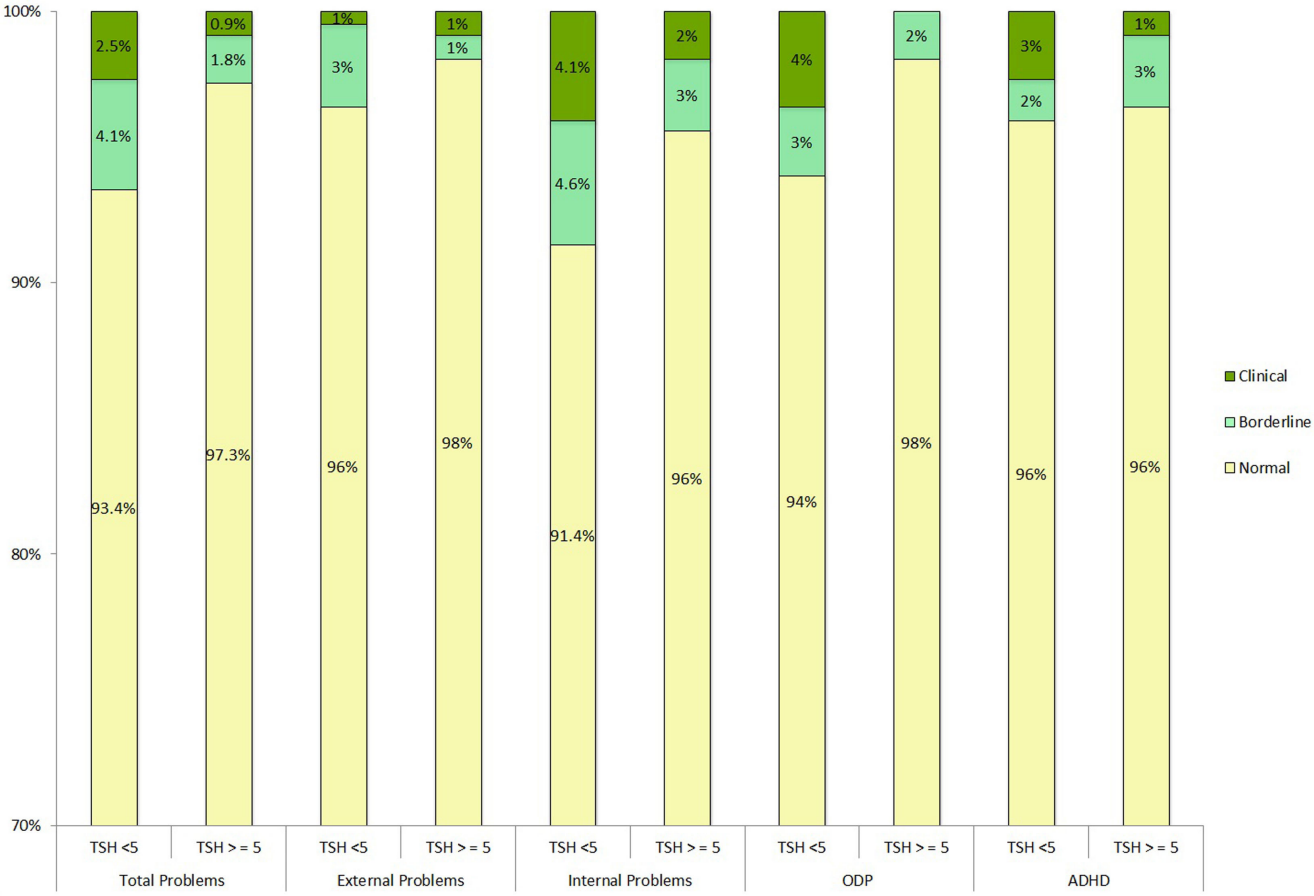
**Neonatal TSH concentration and severity rating at CBCL scores**. PsychoTSH study (*N* = 310), Belgium, 2008–2014. Clinical *T*-scores <65, borderline <65 and <70, and clinical >70. Chi-squared test of independence was non-significant. ODP, oppositional defiant problems; ADHD, attention-deficit hyperactivity disorder; TSH (mUI/L), thyroid-stimulating hormones.

Child Behavior Check List scores in the borderline and clinical range were not more frequently found in the children with elevated TSH compared to those with TSH < 5 mU/L as shown in Figure 1.

### Predictors of CBCL Scores Assessed by Multiple Linear Regression Analysis

The multiple linear regressions of factors explaining variation of CBCL scores at preschool age are shown in Table 5. Elevated TSH concentration at birth was not associated with any CBCL scores. The predictors of higher CBCL scores (meaning higher problems score) were male gender (total problem score, *p* = 0.008; externalizing score, *p* = 0.001), no dietary supplement intake by the child (total problem score, *p* = 0.003; externalizing score, *p* = 0.006), neonatal hospital attendance (total problem score, *p* = 0.023; internalizing score *p* = 0.093), child negative life event occurrence (total problem, score, *p* = 0.008), maternal education level lower than university (total problem score, *p* < 0.0001; externalizing score, *p* < 0.0001; internalizing score *p* < 0.0001), mother’s lifetime smoking behavior up to child birth (internalizing score, *p* = 0.031), maternal suboptimal vitality (externalizing score, *p* = 0.009), and higher maternal mental distress (total problem score, *p* < 0.0001).

**Table 5:** **Multiple linear regression of factors explaining variation of CBCL scores at preschool age**.

	Total problem score (*N* = 269)		Externalizing score (*N* = 282)		Internalizing score (*N* = 289)	
	*R*^2^ = 22%		*R*^2^ = 14%		*R*^2^ = 13%	
	*b* (SE)	*p*-Value	*b* (SE)	*p*-Value	*b* (SE)	*p*-Value
TSH (mU/L)		0.168		0.126		0.104
<5	Ref		Ref		Ref	
≥5	−1.57 (1.14)		−1.71 (1.11)		−2.12 (1.29)	
Gender		**0.008**		**0.001**		^¶^
Female	Ref		Ref			
Male	3.05 (1.15)		3.52 (1.08)			
Mother education level		***p* < 0.0001**		***p* < 0.0001**		***p* < 0.0001**
University or higher	Ref		Ref		Ref	
<University or higher	6.04 (1.28)		4.68 (1.24)		6.42 (1.43)	
Child dietary supplement intake		**0.003**		**0.006**		^¶^
Yes	Ref		Ref			
No	3.53 (1.19)		3.18 (1.15)			
Mother Vitality Index		^¶^		**0.009**		^¶^
Suboptimal vitality			Ref			
Optimal vitality			−4.69 (1.77)			
Maternal mental distress score		***p* < 0.0001**		^¶^		^¶^
Low (<4)	Ref					
Elevated (>4)	6.54 (1.28)					
Smoking during lifetime		^¶^		^¶^		**0.031**
No					Ref	
Yes					2.89 (1.33)	
Neonatal hospital attendance		**0.023**		^¶^		0.093
Yes	Ref				Ref	
No	−4.79 (2.09)				−3.83 (2.27)	
Child negative life events		0.008		^¶^		^¶^
<2	Ref					
≥2	3.22 (1.20)					

*PsychoTSH study (*N* = 310), Belgium, 2008–2014*.

*A higher CBCL score is related to higher developmental problems*.

*^¶^Variables not kept in the models after stepwise backward selection procedure: school attendance, child bilingualism, household incomes, rural/urban habitat, mother’s mental health score, iodine salt consumption, urinary iodine concentration, pregnancy duration, weight at birth, weight gain during pregnancy, mother’s age, season of birth, alcohol during pregnancy, previous miscarriage, breastfeeding, type of delivery, parity*.

*N, number of subject; Ref, referent*.

## Discussion

Our study was the first to investigate the association between the elevation of neonatal TSH level at birth, used as a marker for iodine deficiency during late pregnancy, and the subsequent behavioral development of the offspring. Children with a neonatal TSH above 5 mU/L—used as a marker of iodine deficiency during late pregnancy—were found to have similar internalizing and externalizing behavioral problems scores to children with lower neonatal TSH levels, after adjusting for children and maternal covariates.

Major factors associated with higher CBCL scores (indicative of higher problems) in multivariate analysis in preschool children were: male gender, neonatal hospital attendance, no dietary supplement intake by the child, negative life child events, lower maternal education level, maternal lifetime smoking behavior up to child birth, maternal psychological distress, and maternal low vitality. Previous research has shown that maternal MID and/or mild thyroid dysfunction could lead to behavioral problems in offspring later in life. A prospective study conducted in Sicily, Italy, investigated neuropsychological profiles of 16 children aged 8–10 years old living in a area with moderate iodine deficiency and 11 children in a control group living in an iodine-sufficient area in regards to the thyroid function of their mother during pregnancy (8). They found that 68% of the children showed ADHD symptoms in the iodine deficient area compared to no children in the iodine-sufficient area. In the iodine deficient area, 87.5% of the children born from hypothyroxinemic mothers had ADHD (8). A study conducted on the Dutch Generation R cohort found that maternal hypothyroxineamia during pregnancy was linked to an increased risk of showing ADHD symptoms at 8 years of age (10).

Another study conducted on the Generation R cohort study found that MID during pregnancy, measured by urinary iodine excretion, was related with impaired executive functioning in children of 4 years old (11). Executive functioning involves inhibition, working memory, and organization skills. Impairments in cognitive functioning have been linked to behavioral problems such as ADHD, oppositional defiant disorder, or conduct disorder (11).

In line with the findings of our study, several other studies did not find any relationship between mild thyroid dysfunction potentially due to MID (maternal low fT4) and behavioral problems in the offspring.

A study on the Generation R study cohort found no association between maternal fT4 measured before 18th weeks of pregnancy and total T4 and CBCL behavioral problems scores in children at 1½ and 3 years old (9). On the other hand, they found a relationship between higher levels of maternal TSH and higher externalizing scores in children at 1½ and 3 years old. A study carried out in Finland on 9,479 children aged 8 years old in order to look for an association between maternal thyroid dysfunction and ADHD in the offspring found similar results. No association between maternal low fT4 during pregnancy and ADHD in the offspring was found (32). However, girls whose mother had elevated TSH levels during pregnancy had a higher risk of ADHD (32). In contrast to those findings, a Danish epidemiological study (*N* = 30,295) found no relationship between maternal hypothyroidism and ADHD symptoms in the offspring (33). A higher risk of ADHD in children whose mothers were diagnosed with hyperthyroidism after childbirth (vs. the one diagnosed prior to childbirth) was observed (33).

In the Netherlands, the Generation R study cohort (*N* = 3,139) investigated the association between thyroid peroxidase antibodies (TPOAbs) in healthy pregnant women and child behavior, using the Child Behavior Checklist. A higher risk of behavioral problems and ADHD was found in children whose mothers had elevated TPOAbs during pregnancy (7).

Studies such as ours, which investigate the impact of neonatal TSH levels as an indicator of MID during pregnancy (16–18, 34), only indicate the iodine status during late pregnancy. Subclinical impairment of cognitive and psychomotor development secondary to maternal hypothyroxineamia were found in studies investigating the first half of pregnancy (35–38). In several studies, no adverse outcomes were observed when hypothyroxineamia was studied from midpregnancy (39, 40). However, some studies did not find neurodevelopmental problems in children of mothers with hypothyroxinemia (41) or low urinary iodine excretion measured in the first trimester of pregnancy (42).

The present study was conducted on a large sample stratified by gender and TSH ensuring that the whole range of TSH values below the clinical threshold for the diagnosis of congenital hypothyroidism was included. According to WHO, a proportion of neonates with a concentration of TSH above 5 mUI/L between 3 and 19% indicates MID (12). In Belgium, the frequency of neonatal TSH concentrations above 5 mUI/L fluctuated between 2.6 and 3.3% from 2009 to 2011 (43). This proportion was very low in comparison to results found for Belgian women of childbearing age. Indeed a study has shown that they had a median UIC indicative of MID in the year 2010–2011 (44). However, a median UIC indicative of iodine sufficiency was found in school-aged children (45).

Many factors other than iodine deficiency can influence TSH measured at birth such as the timing of the blood sampling, the use of iodine-containing antiseptics around birth, or exposure to some organochlorines called endocrine disruptors (20, 21). The effect of MID during pregnancy on children behavioral development should thus be complemented by prospective studies using other markers of iodine deficiency during from first trimester of pregnancy, such as maternal UIC or maternal hypothyroxinemia.

Despite the fact that a whole range of variables were collected and taken into account to avoid a confounding effect, it is possible that there is a remaining confounding effect. Other study limitations include its retrospective design, self-reported data, and the fact that behavioral problems were assessed at preschool age rather than at school age (when hyperactive and impulsive symptoms are the more important) and using a self-reported questionnaire rather than a direct psychological assessment by a psychologist.

Future studies aiming to assess MID during pregnancy on child behavioral development should investigate a larger sample, be prospective, and use other markers of iodine deficiency from the first trimester of pregnancy, such as maternal UIC or maternal hypothyroxinemia. Ideally, the behavioral development should be assessed both at preschool and school age.

## Conclusion

The present study was the first to investigate the association between behavioral development of preschoolers using neonatal TSH concentration as a surrogate marker of maternal MID during late pregnancy. No associations were found between neonatal TSH level and children’s behavioral development.

## Ethics Statement

All procedures performed in studies involving human participants were in accordance with the ethical standards of the institutional and/or national research committee and with the 1964 Helsinki declaration and its later amendments or comparable ethical standards. Informed consent was obtained from all individual participants included in the study.

## Author Contributions

All authors contributed to the conception and the design of the study, the data interpretation, and the revision of the article. CT wrote the manuscript, performed the data collection and the data analysis. All the authors approved the final version of the article.

## Conflict of Interest Statement

The authors declare that the research was conducted in the absence of any commercial or financial relationships that could be construed as a potential conflict of interest.

## Abbreviations

ADHD, attention deficit hyperactivity disorder; CBCL, Child Behavior Checklist; DSM, diagnostic and statistical manual of mental disorders; fT4, free thyroxine; MID, mild iodine deficiency; mU/L, micro international unit/liter; T4, thyroxin; TPOAbs, thyroid peroxidase antibodies; TSH, thyroid-stimulating hormone; ULB, Université Libre de Bruxelles; UIC, urinary iodine concentration; WHO, World Health Organization.
